# Pancancer survival prediction using a deep learning architecture with multimodal representation and integration

**DOI:** 10.1093/bioadv/vbad006

**Published:** 2023-01-23

**Authors:** Ziling Fan, Zhangqi Jiang, Hengyu Liang, Chao Han

**Affiliations:** CellEvoX Biotechnology Co. Ltd., Shenzhen, Guangdong 518000, China; Fosun Pharma, Shanghai 200233, China; School of Mathematical Sciences, Shanxi University, Taiyuan, Shanxi 237000, China; CellEvoX Biotechnology Co. Ltd., Shenzhen, Guangdong 518000, China; CellEvoX Biotechnology Co. Ltd., Shenzhen, Guangdong 518000, China

## Abstract

**Motivation:**

Use of multi-omics data carrying comprehensive signals about the disease is strongly desirable for understanding and predicting disease progression, cancer particularly as a serious disease with a high mortality rate. However, recent methods currently fail to effectively utilize the multi-omics data for cancer survival prediction and thus significantly limiting the accuracy of survival prediction using omics data.

**Results:**

In this work, we constructed a deep learning model with multimodal representation and integration to predict the survival of patients using multi-omics data. We first developed an unsupervised learning part to extract high-level feature representations from omics data of different modalities. Then, we used an attention-based method to integrate feature representations, produced by the unsupervised learning part, into a single compact vector and finally we fed the vector into fully connected layers for survival prediction. We used multimodal data to train the model and predict pancancer survival, and the results show that using multimodal data can lead to higher prediction accuracy compared to using single modal data. Furthermore, we used the concordance index and the 5-fold cross-validation method for comparing our proposed method with current state-of-the-art methods and our results show that our model achieves better performance on the majority of cancer types in our testing datasets.

**Availability and implementation:**

https://github.com/ZhangqiJiang07/MultimodalSurvivalPrediction.

**Supplementary information:**

Supplementary data are available at *Bioinformatics* online.

## 1 Introduction

With the development of computational techniques in healthcare, survival prediction has begun playing a pivotal role in improving medical care for patients (Eschrich *et al.*, 2005; Lundin *et al.*, 1999; Montazeri *et al.*, 2016). As a time-to-event prediction, survival time is the main target in survival prediction, which is defined as the length of time from the beginning of observation until death (or another specific event) of the observed patient or until the end of observation (Van Wieringen *et al.*, 2009). Survival prediction investigates the relationship between ultimate clinical outcomes and their closely related influencing factors, from which the death or recurrence of patients can be inferred based on molecular and clinical data. Also, the essential factors mostly affecting final disease outcomes can be recognized similarly to the feature selection process in a machine learning task (Chen *et al.*, 2011; Muthukrishnan and Rohini, 2016).

Kaplan and Meier (1958) first time proposed a non-parametric method for survival prediction. Prior to this work, incomplete observation was a big challenge. Here incomplete observation, also called censored observation, refers to the samples with the event of interest not completely observed (e.g. if a patient withdrew from the study or lost clinical follow up at some point before recurrence or death). This approach, also known as the Kaplan–Meier estimator, is a non-parametric statistical method used to estimate and plot the survival probability as a function of time, where the survival probability at a certain moment is defined as the continued multiplication of the survival ratios at all previous moments. Incomplete observations are considered to be alive in this method. Another classical method for survival prediction is the Cox proportional hazard (CPH) linear model proposed by Cox (1972). This method makes assumptions on the functional form of the effects of covariates (e.g. age, gender and race) on the hazard ratio, and uses a new hazard function for prognosis prediction. Compared to Kaplan–Meier estimator, the CPH model is suitable for not only categorical predictor variables but quantitative variables. Moreover, the CPH model can simultaneously evaluate the impact of multiple risk variables on survival time. Therefore, the CPH model has become a widely used approach in various fields of survival data. Ikeda *et al.* (1991) used the CPH model to analyze the factors affecting the survival time of patients with hepatocellular carcinoma based on clinical data. Recently, with the advent of the neural network, Faraggi and Simon (1995) proposed a non-linear extension to the CPH model. In their method, the classical CPH’s linear predictor is replaced by a non-linear function parameterized by a feed forward neural network. They illustrated the method by using clinical data of patients with prostatic carcinoma. Most recently, this method has been extended to be a more complex structure of the neural network, leveraging modern deep learning (DL) techniques. For instance, Yousefi *et al.* (2017) used Bayesian-optimized deep survival models to analyze high-dimensional genomic data. They demonstrated that the proposed DL method can successfully transmit information across diseases to improve the performance of prognosis prediction. Similarly, Kim *et al.* (2019a) applied the DeepSurv model, a multi-layer feed forward network, on clinical oral cancer data to predict disease outcomes of cancer patients. Their result shows that DeepSurv outperforms the classical CPH method by 8.7% on the testing dataset. More recently, the meta-learning approach, a new transfer-learning method based on neural networks, is proposed by Qiu *et al.* (2020) to tackle the issue of limited sample size for survival prediction. The results show that the meta-learning method achieves better performance compared to regular pre-training and other combined learning methods on three testing cancer datasets when the number of training samples is very limited.

The abovementioned works have focused on only unimodal input, data from a single resource, omitting the opportunity to take advantage of more comprehensive information from other aspects of patients which may influence the overall survival time of patients. A recent study shows that leveraging the multi-omics data of cancer can improve the accuracy compared to single modality for non-small-cell lung cancer subtype classification (Carrillo-Perez *et al.*, 2022). Omics technologies have become increasingly important approaches to obtaining collective characterization and quantification of biological samples (Debnath *et al.*, 2010). In the recent two decades, a large amount of omics data from patients with various diseases has been collected and open-sourced for public research use. For example, The Cancer Genome Atlas (TCGA) (Malta *et al.*, 2018), a multi-institutional project, has collected standardized clinical, imaging and multi-omics data for a wide range of cancer types. While previous works have explored methods for survival prediction using single data type (Faraggi and Simon, 1995; Ikeda *et al.*, 1991; Kim *et al.*, 2019a; Zhu *et al.*, 2016), very few methods, in recent years, have been proposed fully taking advantage of multiple data modalities (Carrillo-Perez *et al.*, 2022).

One way to address the issue is via multimodal integration (Boehm *et al.*, 2022; Garali *et al.*, 2018). Multimodal integration is a concept of fusing data and information from multiple modalities with the goal of enhancing prediction performance compared to merely depending on a single modality (Baltrušaitis *et al.*, 2019). The approaches for integrating omics data emerging in recent years are not only innovative but also have transferability for use in various disciplines. Dimension reduction combined with a weighted average fusion strategy is a simple way to fuse multi-omics data (Rappoport and Shamir, 2019; Tan *et al.*, 2020). Feng *et al.* (2021) applied kernel Principal Component Analysis (Schölkopf *et al.*, 1998) to extract features for gene expression profile, isoform expression profile and DNA methylation data, and then convert them into three similarity matrices. Next, they took the average of these matrices as a fused matrix and applied it to a spectral clustering algorithm for cancer subtype discovery. Qi *et al.* (2021) proposed a sparse canonical correlation analysis for cancer classification where each single omics data is first projected into a unified space and then integrated by weighted averaging strategy. Multiple kernel learning (MKL) is an extension to kernel support vector machine (SVM) which has received great popularity in the omics integration field (Gönen and Alpaydin, 2011). MKL computes a kernel for each dataset and integrates them into a similarity matrix that describes patients across all multi-omics datasets. For example, Zhang *et al.* (2016) combined SimpleMKL, which is based on weighted *L*_2_-norm regularization, with minimum redundancy feature selection method integrating inputs from five different omics datasets. This approach improves the accuracy of survival prediction to 94.8% compared to 87.9% by SVM on the Glioblastoma Multiforme dataset. With improved interpretability, the pathway-based integration method has been wildly adopted to discover the connection between multi-omic data with phenotypic outcomes (Jeong *et al.*, 2015; Kim *et al.*, 2014, 2019b). Kim *et al.* (2018) combined the directed random walk method and denoising autoencoder to integrate the pathway information of gene expression and DNA methylation over an integrated gene-gene graph on a breast cancer dataset and obtained improved survival prediction performance. Neural Networks are another family of popular models for multimodal fusion. Due to their ability to provide end-to-end training in both the multimodal representation component and the fusion component, they have received considerable attention in recent years. Fan *et al.* (2020) proposed MOTA, a network-based approach, integrating multi-omics data to rank candidate disease biomarkers for biomarker discovery. They tested MOTA on three sets of multi-omics data representing three cohorts of hepatocellular carcinoma cases for ranking disease-associated molecules and the results show that MOTA allows the identification of more top-ranked biomarkers compared to traditional statistical methods. OmiEmbed, proposed by Zhang *et al.* (2021), is a compound-task DL architecture based on deep embedding and downstream task modules. In terms of cancer type classification, the method achieves a higher F1-score (0.9683 ± 0.0020) among 33 cancer types by integrating RNA-Seq gene expression, DNA methylation and miRNA expression compared to a single modality. To utilize the correlations between gene expression modal and DNA methylation modal over time, an adaptive multimodal integration method was proposed by using bidirectional long short-term memory and ordinal Cox model network recently (Bichindaritz *et al.*, 2021; Bichindaritz and Liu, 2022). In other studies, the convolutional neural network was used to integrate features from medical images to improve prediction performance (Ning *et al.*, 2020; Silva and Rohr, 2020).

The survival prediction based on multimodal integration task is more unstructured and thus more challenging than traditional DL tasks as reviewed previously. The multimodal integration not only has to deal with correlations between different data types but also needs to tackle the superposition of noise from different modalities (Gevaert *et al.*, 2012). Unsupervised learning has been found to address the issues in recent work (Fan *et al.*, 2018). Inspired by the Siamese network, Cheerla and Gevaert (2019) modified the contrastive loss formula ($L(W,Y,x_{1},x_{2})=(1-Y)\frac{1}{2}D_{W}^{2}+Y\frac{1}{2}{\left[\text{max}(0,M-D_{W})\right]}^{2}$, where *W* is the network weight, $x_{1}$ and $x_{2}$ are the pair-wise inputs, and Y is the paired label (if $x_{1}$ and $x_{2}$ belong to the same class, *Y *=* *0, otherwise *Y *=* *1). *D_W_* represents the Euclidean distance between $x_{1}$ and $x_{2}$ in the variable space. Note that the parameter *M* controls the degree of ‘rejection’ between clusters), and developed an unsupervised learning model for multimodal representation. Their work shows that adopting unsupervised learning to process multiple different data types can effectively integrate information from multimodal data and provide robust representation for prediction. Another work, Droniou *et al.* (2015) proposed an unsupervised network architecture to address the problem of multimodal learning for autonomous robots. Their results indicate that unsupervised representation techniques allow us to exploit the correlations between data modalities. In this work, we proposed a multimodal integrative DL architecture built on unsupervised representation learning and attention mechanism techniques for survival prediction. Our model learns high-level representations for multimodal data, including clinical data and several high-dimensional molecular data modalities, using unsupervised learning. The attention-based method is applied to fuse the learned multimodal representations and the fused vector is then fed into a fully connected neural network to predict the hazard ratio for cancer patients. First, we evaluated our model on different combinations of data modalities for pancancer prediction and the evaluation results show that our model performs best with the combination of clinical and mRNA modalities. Next, we experimentally demonstrated that training on pancancer data can improve the performance of survival prediction. Finally, we tested the proposed approach on twenty cancer types and the results show that our model outperforms the previous work (Cheerla and Gevaert, 2019) on most cancer types. The implementation of the proposed method is available in GitHub.

## 2 Methods

### 2.1 Datasets and data preprocessing

In this study, we adopted four different data types, i.e. clinical data, gene expression (mRNA) data, microRNA expression (miRNA) data and gene copy number variation (CNV) data. The clinical data, mRNA data and miRNA data were downloaded from the PanCanAtlas TCGA project (Public accessible at Genomic Data Commons), where more than 11 000 patient samples across 33 tumor types (Hutter and Zenklusen, 2018; Malta *et al.*, 2018) were collected. The clinical dataset describes the clinical information and annotations of 11 160 cancer patients. In our study, five clinical variables were used: cancer type, gender, race, histological type and age, as those were used by Silva and Rohr (2020). The miRNA dataset contains 743 miRNA feature records, and the mRNA dataset contains RNA-Seq counts for 20 531 mRNAs. The CNV data were downloaded from University of California Santa Cruz (UCSC) Xena (UCSC Xena: http://xena.ucsc.edu/public/) (Goldman et al., 2019). The CNV dataset consists of numeric CNV values for 24 776 genes estimated by the GISTIC2 (Genomic Identification of Significant Targets in Cancer, version 2.0) method (Mermel *et al.*, 2011).

For preprocessing, we first excluded patients with missing data and/or follow up time in the clinical dataset. To reduce the computational cost, the variance threshold method was applied to select the features with variance, calculated over all patients, higher than the given threshold (Fida *et al.*, 2021; Silva and Rohr, 2020). We conducted a preliminary study to determine the threshold of the variance (results are shown in Supplementary Material S1) and the thresholds 7 and 0.2 were selected for mRNA and CNV modalities, respectively, in our study. As a result, 1579 mRNA features, and 2711 CNV features were maintained in our study. Then, all continuous variables were scaled into the interval from 0 to 1 by min-max normalization (Gajera *et al.*, 2016). Since the examination and treatment methods for each cancer are different, each sample in the dataset may not have all the modalities required for the experiment so we replaced the missing modalities of the sample with all zero vectors. Table 1 describes the data information after preprocessing in detail.

**Table 1.** Summary information of the different modalities after preprocessing

Modality	No. of patients	No. of features		PercentageAll zero vector (%)
		Continuous	Categorical	
Clinical	11 094	1	4	–
miRNA	11 094	743	–	9.7
mRNA	11 094	1579	–	3.1
CNV	11 094	2711	–	8.3

In this study, the pancancer datasets were split into training, validation and testing sets in 60-20-20 ratio. The 5-fold cross-validation method was adopted to evaluate the model performance.

### 2.2 Model architecture

**Fig. 1.**
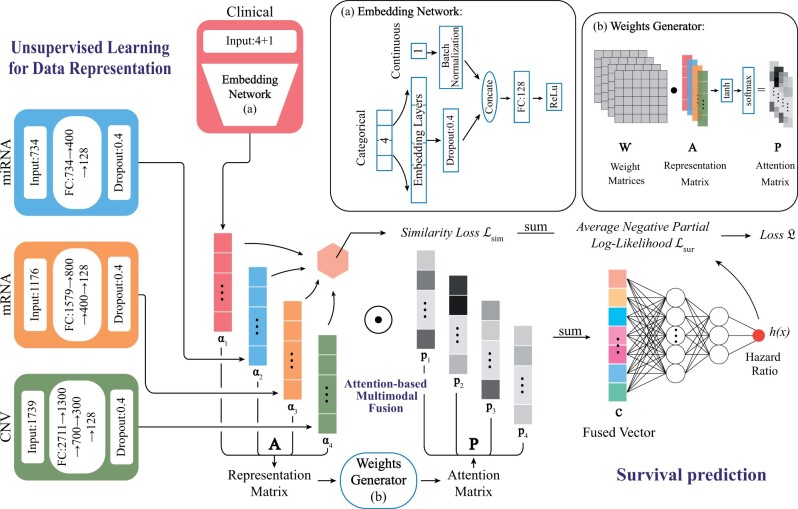
The architecture of the proposed model. Each modality adopts a different neural network setup to learn its representation from its original input. For the clinical data, we use embedding layers with dropout [visualized as Embedding Network (**a**)]. For the miRNA, mRNA and CNV data, we use FC layers with BN and ReLu activation after each layer. These setups extract representations, with a fixed length, which are then fed into Weights Generator (**b**) to generate attention weights and aggregated into a fused vector for survival prediction

As shown in Figure 1, our proposed model structure consists of three parts, namely: unsupervised learning for data representation part, attention-based multimodal fusion part and survival prediction part. In the unsupervised learning part, we started with applying different neural network setups with fully connected (FC) layers for different modal types to extract representations of original data. For instance, we adopted categorical embeddings to tackle four categorical variables in the clinical dataset from the TCGA database. The unsupervised learning part is inspired by the Siamese network (Chopra *et al.*, 2005) which uses the similarity between two different views of objects to create representations. Similarly, in our work, the relationship between the modalities of patients was exploited using this idea to extract representations more effectively. In the part of multimodal fusion, the attention-based fusion method was used to integrate the representations of different modalities into a compact vector. Finally, in the survival prediction part, the fused vector was fed into a neural network to compute the hazard ratio for survival prediction. The three parts mentioned above will be described in detail in the next three sections.

### 2.3 Unsupervised representation learning

Due to the flexibility and competitive performance, the neural network with FC layers has been wildly applied to extract representations recently (Silva and Rohr, 2020). In our work, different neural network setups with FC layers were used for representation extraction from the original numerical data of different modalities. In addition, the Batch Normalization (BN) method was adopted to accelerate neural network training by normalizing the layer’s input (Ioffe and Szegedy, 2015). For the clinical data, we used categorical embedding layers for the four categorical variables, namely, cancer type, gender, race and histological type with dropout to encode the categorical variables into a numerical vector. The continuous variable (i.e. age), normalized by BN, was then concatenated with the numerical vector and the concatenated vector was fed into an FC layer for fixed representation length. For miRNA, mRNA and CNV modalities, we used two to four FC layers with ReLu activation and BN following each layer to extract representations with fixed length. Then, we concatenated the representation vectors $\alpha_{1},\alpha_{2},\ldots ,\alpha_{n}\in R^{m\times 1}$ from modality-specific setups to obtain a representation matrix $A\in R^{m\times n}$ composed of *n *=* *4 representations representing the number of different data modalities and the value of the fixed length *m* equal to 128.

After extracting representations from original data of different modalities, we further adopted an unsupervised learning approach for a higher-level feature representation learning from modalities. Cheerla and Gevaert (2019) proposed an unsupervised learning method to exploit the relationship between different data modalities using a loss function modified from the Siamese network. In their work, the similarity between representations from different modalities of a single patient is maximized and the similarity between representations from the same modality of different patients is minimized. However, the loss function in Cheerla and Gevaert (2019) computes the accumulative loss between every pair of patients in a batch which will result in an exponential training time and a huge amount of computation when training the model. In our work, we modified their loss function with the purpose of avoiding the beforementioned weakness. To do so, we proposed a loss function where samples in a batch will be matched randomly in pairs, and then the loss was computed only between the matched pairs. To confirm that our method reduces training time and computational cost without hurting the model performance, we conducted an experiment where we compared the performance of the model using our proposed loss and the loss used in Cheerla and Gevaert (2019). The result (shown in Supplementary Material S2) suggests that our training method not only reduces the computational complexity but hardly affects the final performance of the model. The loss $L_{\text{sim}}$ is defined as in Equations (1)–(4):

(1) $$\text{sim}(x,y)=\sum_{1\leq i\leq n}\frac{\alpha_{i}(x)\cdot \alpha_{i}(y)}{\left||\alpha_{i}(x)||||\alpha_{i}(y)|\right|},$$

(2) $$\text{sim}(x,x)=\sum_{1\leq i<j\leq n}\frac{\alpha_{i}(x)\cdot \alpha_{j}(x)}{\left||\alpha_{i}(x)||||\alpha_{j}(x)|\right|},$$

(3) $$L(x,y)=\text{max}\{0,M+\text{sim}(x,y)-\frac{1}{2}\text{sim}(x,x)-\frac{1}{2}\text{sim}(y,y)\},$$

(4) $$L_{\text{sim}}=\sum_{x,y}L(x,y),$$

where $\alpha_{i}(x)$ denotes the *i*th modality representation of patient *x*. Here, the purpose of M is to effectively avoid model collapse. When *M* is higher, the representation vectors of a patient will be tighter; on the contrary, the vectors will be more discrete. Hence, finding an appropriate *M* can make the loss function play a greater role. We selected *M *=* *0.2 empirically in this work.

### 2.4 Attention-based data fusion

Because different modalities have different degrees of importance for the survival prediction of cancer, we adopted an attention-based approach to estimate the weights reflecting the importance of each modality. The attention-based multimodal fusion part takes the representation matrix **A** described earlier as input. During training, the model will learn an attention matrix $P\in R^{m\times n}$, with each column $p_{j}$ providing the weights for the corresponding feature vector $\alpha_{j}$. To produce a compact representation vector $c={\left(c_{1},c_{2},\ldots ,c_{m}\right)}^{T}$, we multiply the corresponding elements of matrix **A** and **P**, and then sum the rows:

(5) $$s_{j}=\text{tanh}(W_{j}\cdot \alpha_{j}),\;j\in \{1,2,\ldots ,n\},$$

(6) $$P=\text{softmax}((s_{1},s_{2},\ldots ,s_{n})),$$

(7) $$c_{i}=\sum_{j=1}^{n}p_{ij}\alpha_{ij},\;i\in \{1,2,\ldots ,m\},$$

where $W_{j}\in R^{m\times m}$ denotes the *j*th weight matrix of a tensor $W\in R^{n\times m\times m}$ and the softmax function is applied on each row. Intuitively, the model can learn the importance of the modalities and assign different attention for integration. To a certain extent, this method can effectively reduce the influence of the noises carried by the modalities and amplify valid information.

### 2.5 Survival prediction

Hazard ratio is the essential concept in survival prediction which objectively reflects the risk of a patient at a time point. The main goal of survival prediction model learning is to learn a function adopting an appropriate parametrizing approach for hazard ratio estimation. The CPH model (Cox, 1972) defines the half-parameter hazard function where a linear function is used as a predictor. Instead of using the linear predictor function, we applied a non-linear hazard function *h*(*x*) parameterized by the weights of a neural network in our proposed model. The fused vector **c** described in the previous section is fed into a two-layer fully connected neural network with ReLu activations and finally a single node to output the calculated hazard ratio. To accurately predict the prognosis, we used the average negative partial log-likelihood as the loss function $L_{\text{sur}}$ to train the model. The function is defined as:

(8) $$L_{\text{sur}}=-\frac{1}{N}\sum_{i:E_{i}=1}\left(h(x_{i})-\text{log}\;\sum_{j:T_{j}\geq T_{i}}e^{h(x_{j})}\right),$$

where *T_i_* and *E_i_* respectively represent the event time and event indicator. The number of non-censored patients (with *E_i_* = 1) is represented by *N* and $x_{i}$ is the input data for the *i*th patient. Due to its superior sensitivity to the difference between true labels and predicted by the model, it has been widely used in recent studies (Mobadersany *et al.*, 2018; Yousefi *et al.*, 2017). Finally, combing the unsupervised model, the final loss function L becomes:

(9) $$L=L_{\text{sur}}+\lambda L_{\text{sim}},$$

where the parameter *λ* is the balance factor between $L_{\text{sur}}$ and $L_{\text{sim}}$. In our experiments, we simply fixed the *λ* to 0.3.

### 2.6 Visualization

To illustrate the effect of the proposed unsupervised learning part visually, we visualized the data as follows: we first used the principal component analysis (PCA) to map feature vectors with 128 in length into 2D space, and then drew the PCA results to show the effect of the unsupervised representation learning. PCA is a commonly used dimension reduction approach that compresses high-dimensional vectors to lower dimensions while keeping most of the original information (Karamizadeh *et al.*, 2020).

### 2.7 Evaluation index

In this study, we evaluated our model performance using the concordance index (C-index), which is one of the most widely used metrics to evaluate the survival predictions of censored survival data (Harrell *et al.*, 1982; Schmid *et al.*, 2016). The C-index quantifies the proportion of concordant pairs in the total number of possible evaluation pairs as:

(10) $$C-\text{index}=\frac{\sum_{i\neq j}1(h(x_{i})<h(x_{j}))1(T_{i}>T_{j})E_{j}}{\sum_{i\neq j}1(T_{i}>T_{j})E_{j}},$$

where 1 is the indicator function of whether the expression in parentheses is true or false. The C-index ranges from 0 to 1. The closer the C-index to 1, the closer the prediction order to the real one; the closer the C-index to 0.5, the prediction of the model is closer to random prediction. Since the C-index emphasizes the order of predictions rather than the correctness of the prediction of one sample alone, it is useful for estimating proportional hazard models.

## 3 Results

### 3.1 Unsupervised learning representation for multimodal data

**Fig. 2.**
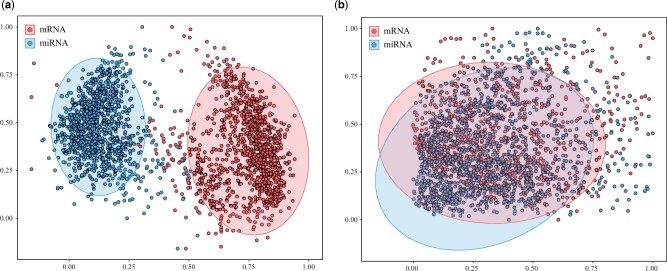
The visualization of multimodal representations for ∼1100 breast cancer patients with the miRNA and mRNA modalities. The 128-length representation vectors produced by network setups were compressed using PCA into 2D space. The representation vectors from the same modality are more separated and the representation vectors from different modalities are more mixed, after the unsupervised learning part

The main purpose of the unsupervised learning part is to extract useful representations for input data and reduce the noise present in the datasets, so as to make full use of the information of each modality. We explored the unsupervised learning part of our model by visualizing the representation vectors from the mRNA and the miRNA modalities of breast cancer, as shown in Figure 2. On one hand, the comparison of Figure 2a and b shows that the unsupervised learning part can make the representation vectors from the same modality more separated. Intuitively, the scattered representation vectors magnify the degree of discrimination between different patients so that the information of a single modality can be exploited more effectively. On the other hand, Figure 2 shows that the representation vectors from different modalities are more mixed together after the unsupervised learning part, indicating that the unsupervised learning learns the correlation between different modalities.

### 3.2 Evaluation of model architecture

To verify the effectiveness of the architecture proposed in this work, we tested the influence of the unsupervised learning and multimodal fusion parts on the prediction performance, respectively, while maintaining the integrity of the proposed framework. The clinical data and CNV data were selected as input for the testing. In the experiment using the unsupervised learning part alone, we fixed the intermediate vector $s_{j}$ in formula (5) as an all-ones vector to force attention to be evenly distributed across modalities. In terms of using the attention fusion part only, we removed the unsupervised loss $L_{\mathrm{sim}}$ from the proposed loss function to test whether the unsupervised learning part enhances the final prediction performance. The results are shown in Table 2. According to the table, the performance of the proposed structure is better than when the two parts are used independently (C-index of 0.755 versus 0.740 for the only unsupervised model and 0.750 for the only fusion model), which confirms the effectiveness of our structure.

**Table 2.** The performance of the unsupervised learning module and fusion module on multimodal data, including clinical and CNV, when used individually and in combination

Modal	Only unsupervised learning	Only fusion	Combined (proposed method)
C-index	0.740	0.750	**0.755**

The bold entries represent the highest C-index results.

### 3.3 Survival prediction using pancancer training dataset

Next, we used the proposed model for survival prediction using only a single modality and multiple modalities as input. The performances of our model, evaluated by 5-fold cross-validation, are shown in Table 3. The average C-index of the model on only a single modality is 0.729, while the average C-index of the model on multiple modalities is 0.771. The result shows that using multimodal data as input for prediction is generally better than using only single modal data. This finding may confirm that using multimodal data can provide more complete and efficient information for survival prediction compared to using single modality (Carrillo-Perez *et al.*, 2022). The performance of the proposed model with single modality input suggests that mRNA is the most informative modality, closely followed by clinical, while CNV is the least informative. In terms of multimodal input, we find that the highest C-index is achieved with the combination of clinical and mRNA data, while using all four modalities our model achieves a lower C-index (C-index of 0.780 versus 0.772). Moreover, we observed that the predictive performance of the proposed model degrades when adding the CNV modality.

**Table 3.** Model performance on mixed cancer types using single modal and multimodal training datasets by the measurements of C-index (along with their standard deviations)

Modality	C-index				Mean (proposed model)
	CPH	RSF	MultiSurv	Proposed model	
Clin	0.648(±0.010)	0.746(±0.008)	0.739(±0.021)	**0.757**(±0.008)	0.729
mRNA	0.715(±0.013)	0.740(±0.013)	0.752(±0.012)	**0.766**(±0.007)	
miRNA	0.714(±0.010)	**0.735**(±0.005)	0.726(±0.006)	0.734(±0.014)	
CNV	0.589(±0.008)	0.627(±0.007)	0.649(±0.006)	**0.659**(±0.011)	
Clin+CNV	–	–	0.751(±0.009)	**0.755**(±0.009)	0.771
Clin+mRNA	–	–	0.766(±0.009)	**0.780**(±0.006)	
Clin+miRNA	–	–	0.758(±0.011)	**0.769**(±0.009)	
Clin+mRNA+CNV	–	–	0.765(±0.015)	**0.775**(±0.007)	
Clin+miRNA+CNV	–	–	0.760(±0.009)	**0.766**(±0.017)	
Clin+miRNA+mRNA	–	–	0.769(±0.009)	**0.779**(±0.009)	
Clin+miRNA+mRNA+CNV	–	–	0.769(±0.015)	**0.772**(±0.010)	

Clin, clinical data; mRNA, gene expression data; miRNA, microRNA expression data; CNV, gene copy number variation data; CPH, Cox proportional hazards; RSF, random survival forest; MultiSurv, proposed by Silva and Rohr (2020). The bold and underline entries represent the highest and the second highest C-index results, respectively.

Furthermore, we investigated using different combinations among clinical, mRNA and CNV, to test whether different combinations of modalities will affect the survival prediction of single cancer. We selected the same twenty cancer types as those used by Cheerla and Gevaert (2019) in our study, where patients have significantly different survival patterns. The results are shown in Table 4. We found that using clinical data alone as input outperforms the other combinations on only two cancer types (KICH and OV). For eight cancer types, integration of clinical, mRNA and CNV gives the best performance with the most striking example THCA (C-index 0.953). For ten cancer types, the model integrating clinical and mRNA is the best. The results suggest that the combination of modalities has a great impact on the final prediction. For KIRP, the difference in model performance, measured by C-index, between using only clinical data and using the combined data of clinical and mRNA modalities reached 0.33. The results also validate that the mRNA modality is essential for the prognosis prediction of the studied cancer types in our study.

**Table 4.** Model performance on individual cancer type, using multimodal training datasets

Cancer type	No. of patients (alive)	Clin	Clin+mRNA	Clin+mRNA+CNV
BLCA	411 (231)	0.598	0.636	**0.665**
BRCA	1096 (945)	0.605	**0.681**	0.665
CESC	307 (236)	0.552	**0.703**	0.676
COADREAD	628 (500)	0.583	**0.596**	0.595
HNSC	527 (304)	0.563	0.641	**0.642**
KICH	112 (100)	**0.716**	0.663	0.714
KIRC	537 (360)	0.589	**0.721**	0.708
KIRP	290 (246)	0.489	**0.817**	0.791
LAML	186 (66)	0.681	**0.695**	0.674
LGG	514 (389)	0.753	0.817	**0.818**
LIHC	376 (244)	0.521	**0.639**	0.627
LUAD	513 (329)	0.529	0.636	**0.638**
LUSC	498 (283)	0.552	**0.598**	0.579
OV	582 (234)	**0.601**	0.591	0.584
PAAD	185(85)	0.577	**0.637**	0.617
PRAD	500 (490)	0.524	**0.626**	0.576
SKCM	455 (241)	0.605	0.644	**0.648**
STAD	437 (268)	0.555	0.568	**0.581**
THCA	507 (491)	0.921	0.943	**0.953**
UCEC	547 (456)	0.655	0.704	**0.707**

*Note*: Cancer types are defined according to TCGA cancer codes.

The bold entries represent the highest C-index results.

### 3.4 Comparison between using single cancer and pancancer training datasets

**Fig. 3.**
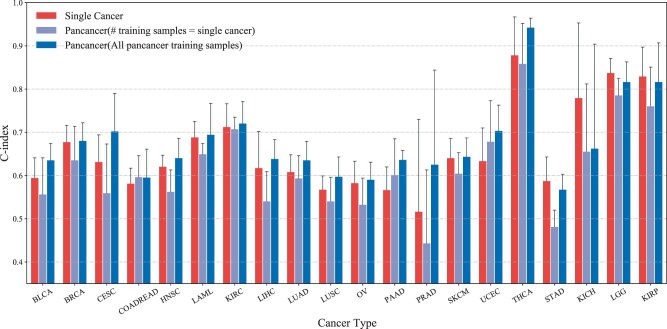
C-index scores of the proposed model trained by single cancer and pancancer datasets on twenty cancer types using clinical and mRNA modalities

Next, we tested whether the use of pancancer data will improve the survival prediction accuracy for individual cancer types. We first compared the performance of the models trained on single cancer data and that of the models trained on pancancer data with all training samples. For single cancer experiments, we selected patients with the same cancer type in pancancer training-validation-test sets to form the single cancer training-validation-test sets. It is worth noting that for each cancer type the models trained on the pancancer data and the single cancer data were tested on the same test set in one fold. Clinical and mRNA modalities were used in this experiment and the results are shown in Figure 3. Original results (numerical value) are shown in Supplementary Material S3. Except for STAD, KICH, LGG and KIRP, these four cancer types, the models trained using pancancer datasets with all training samples outperform the models trained using single cancer datasets. This result shows that the performance of survival prediction for individual cancer types can be improved by using the pancancer dataset as the training dataset.

In the previous set of comparisons, we did not guarantee the number of training samples is the same. Next, we wanted to further explore whether the models trained on pancancer data are still better when the number of training samples is the same. Therefore, we conducted another set of comparisons where we only use pancancer (mixed cancer types) data with the same number of training samples as single cancer data. The lavender bar represents the set of experimental results in Figure 3. Figure 3 shows that the performance of the models trained using single cancer generally outperforms those trained using pancancer with the same number of training samples. The results indicate that although there are commonalities between cancers, single cancer itself also has special characteristics. Therefore, when the number of training samples is the same, the performance of the model trained with data from mixed cancer types is, as expected, not as good as the performance of the model trained with only the single target cancer type.

### 3.5 Comparison with previous work

Firstly, we evaluated our proposed model with single modality inputs in comparison to three other methods: CPH, Random Survival Forests (RSF) and the attention-based MultiSurv method (Silva and Rohr, 2020), on pancancer datasets using 5-fold cross-validation. For CPH and RSF methods, the variance threshold method was adopted to further reduce the data dimensionality, selecting only 100 features with the highest variance. As shown in Table 3, for all of the single modalities, but miRNA, the proposed model achieves the highest C-index. These results validate the individual neural network setup for feature extraction used in our model. Secondly, we compared the MultiSurv and our proposed method using multimodal data, with different combinations of data modalities. The results are shown in Table 3 which show that the proposed model outperforms the MultiSurv in all of the combinations of data modalities.

**Fig. 4.**
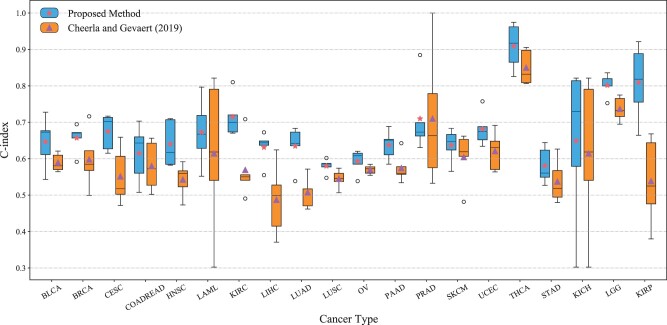
C-index of the proposed model and the previous work (Cheerla and Gevaert, 2019) on the twenty cancer types using the modality combination of clinical, miRNA and mRNA. The proposed model outperforms the previous work on 19 cancer types

Finally, we conducted a comparison study between our proposed model with the state-of-the-art DL-based model (Cheerla and Gevaert, 2019) for single cancer prediction using pancancer datasets. To conduct this comparison, clinical, miRNA and mRNA modalities were used. The results are shown in Figure 4. For each cancer type, the mean score of the proposed model and those of Cheerla and Gevaert (2019) are represented by red stars and purple triangles, respectively. Another set of comparison results using clinical, miRNA and CNV modalities are shown in Supplementary Material S4.

As shown in Figure 4, our proposed method achieves a higher C-index compared to Cheerla and Gevaert (2019) in the majority of cancer types (19 out of 20). Out of the nineteen cancer types, our proposed model performs much better than Cheerla and Gevaert (2019) on six cancer types (the difference in C-index is more than 0.1).

## 4 Discussion

Previous studies have shown that leveraging multimodal data of cancers can enhance the performance of survival prediction compared to merely using a single modality (Zhang *et al.*, 2021). However, the survival prediction based on multimodal integration task is more unstructured and thus the performance of DL is still limited. This study aimed to propose a DL architecture with multimodal representation and integration based on unsupervised learning and attention mechanism for survival prediction.

According to the performance of the proposed model with multimodal input, we observe that our model performed best on the combination of clinical and mRNA modalities, instead of using all modalities. A similar pattern of results is obtained in Silva and Rohr (2020). Our speculation for this phenomenon is that the increased data when adding more data modalities may lead to a higher risk of overfitting. Another interesting finding is that the prediction performance of the proposed model degrades when adding the CNV modality. We speculate that the CNV modality may contain more irrelevant information to the survival prediction task which may introduce unwanted noise instead of providing useful information for prediction. As mentioned in Valle *et al.* (2022), those ‘hitchhiker’ genes accounting for the majority of CNV dataset may barely provide information beneficial to the final prediction performance.

We conducted two sets of comparative experiments to further understand the impact of pancancer data. We find that the large sample size in pancancer data may be the direct factor contributing to the improvement of the performance of the model. It seems that when the data size of pancancer data is large enough, the positive effect caused by the increase in data size will exceed the adverse effect of noises introduced by the mixed cancer types. However, there are several types of cancer in which the use of pancancer data has an adverse effect (e.g. STAD and KICH). This may be due to the fact that these cancers have very unique characteristics so that information from other cancers may not help with survival prediction. In other words, the use of pancancer data will significantly improve the performance of the model if the remaining cancer data contains strong association information to the target cancer. We believe that the strong association of each target cancer to the remaining cancers in pancancer data is the fundamental reason for pancancer data help to achieve good model performance. In fact, some cancers have been confirmed to be related to each other and have inherent interdependencies at the molecular level (Cancer Genome Atlas Network, 2012).

We compared the performance of our method to the previous work (Cheerla and Gevaert, 2019) across twenty cancer types using the modality combination of clinical, mRNA and miRNA. We observe that our model generally shows better performance than the previous work. The performance of our model can be explained by the observations from Table 4 that the combinations of modality can have a great influence on the final survival prediction performance. Compared to the average fusion method used in the previous work, we apply a more flexible attention-based fusion approach so that our model can automatically adjust the weights according to the importance of modalities. Moreover, our proposed fusion method also allows our model to reduce the negative impact of noise, carried by different modalities, on survival prediction. In terms of the training cost, our model uses a modified loss function and is trained by the random match strategy to reduce computational cost and the results indicate that such modifications can lead to a substantial reduction in training cost without hurting the model performance.

In conclusion, the proposed DL architecture, based on unsupervised learning and attention mechanism, can effectively integrate diverse information from different modalities to accurately predict the prognosis of patients. According to the results, the performance of our model performs better compared to previous work to act as a powerful tool for future studies. In future work, we will focus on developing special neural network setups to extend the model to integrate more diverse sources of data to further improve predictive performance. Furthermore, we intend to apply the multimodal DL architecture to other tasks, such as cancer subtypes classification and marker discovery.

## Supplementary Material

vbad006_Supplementary_Data

## Data Availability

The data underlying this article are available in Zenodo, at https://zenodo.org/record/7579405.
