# Supplementary material for: Pancancer survival prediction using a deep learning architecture with multimodal representation and integration
Source: Bioinform Adv. 2023 Jan 23;3(1):vbad006. doi: 10.1093/bioadv/vbad006 (PMC9945067; doi:10.1093/bioadv/vbad006)
Supplement: vbad006_Supplementary_Data [file vbad006_supplementary_data.docx]

**Supplementary Material**

Pancancer survival prediction using a deep learning architecture with multimodal representation and integration

This Supplementary Material consists of the following:

- Section S1. Data Preprocessing
- Section S2. Evaluation of the proposed loss function
- Section S3. Numerical values for Figure 3
- Section S4. Comparison with Cheerla and Gevaert (2019) using the modality combination of clinical, miRNA, and CNV

**Section S1. Data Preprocessing**

Variance threshold method is an efficient filter approach for feature selection which assumes that the features with a higher variance may contain more useful information. In our study, we first calculated the variance value of each feature in a dataset over all samples. Then, we trained the proposed model to determine the approximate threshold value according to the predictive performance (C-index) and computational costs (training time). The results are shown in **Table S1**. Finally, we removed the features with a variance lower than the determined threshold.

| **Table S1.** The C-index and training time of the proposed model on mRNA data and CNV data preprocessed with different threshold values. | | | | |
| --- | --- | --- | --- | --- |
| Modality | Threshold value | # Features | C-index | Time |
| mRNA | 6 | 2181 | 0.763 | 13.2min |
|  | **7** | 1579 | 0.766 | 11.9min |
|  | 8 | 1176 | 0.765 | 11.1min |
|  | 9 | 880 | 0.762 | 10.5min |
| CNV | 0.18 | 3877 | 0.641 | 20.6min |
|  | **0.2** | 2711 | 0.641 | 14.3min |
|  | 0.25 | 1162 | 0.624 | 11.1min |
|  | 0.3 | 613 | 0.618 | 10.1min |

**Section S2. Evaluation of the proposed loss function**

Since implementing the loss in Cheerla and Gevaert (2019) to train the model requires large amounts of training time and computational cost, we compared different times of random matches when calculating the loss in Cheerla and Gevaert (2019). We selected the modality combination of clinical and miRNA in this experiment, and the results are shown in **Table S2** When the times of random matches reach twelve, the required training time reaches 5.80 hours and the C-index barely increases so we stopped the experiment here. This result shows that although the number of random matches gradually increases, the performance of the model does not improve correspondingly. This phenomenon suggests that our proposed loss calculation not only will not adversely affect the prognosis prediction accuracy but also saves training time and computational cost compared to the original loss in Cheerla and Gevaert (2019).

| **Table S2.** The C-index and training time of the model with different number of random matches. | | | | | | |
| --- | --- | --- | --- | --- | --- | --- |
| # random matches | 1 | 3 | 5 | 7 | 10 | 12 |
| Training time | 0.57h | 1.48h | 2.42h | 3.40h | 4.67h | 5.80h |
| C-index | 0.7688 | 0.7690 | 0.7690 | 0.7688 | 0.7682 | 0.7692 |

**Section S3. Numerical value for Figure 3**

The original numerical values in Figure 3 are shown in **Table S3**.

| **Table S3.** Numerical value of the C-index (along with their standard deviations) for Figure 3 | | | |
| --- | --- | --- | --- |
| Cancer site | Single cancer | Pancancer  **(# of training samples = single cancer)** | Pancancer  **(All pancancer training samples)** |
| BLCA | 0.595(0.046) | 0.557(0.084) | **0.636**(0.038) |
| BRCA | 0.678(0.038) | 0.636(0.078) | **0.681**(0.041) |
| CESC | 0.632(0.062) | 0.560(0.113) | **0.703**(0.087) |
| COADREAD | 0.582(0.035) | **0.597**(0.049) | 0.596(0.065) |
| HNSC | 0.621(0.026) | 0.563(0.050) | **0.641**(0.045) |
| LAML | 0.689(0.036) | 0.650(0.024) | **0.695**(0.072) |
| KIRC | 0.713(0.053) | 0.708(0.027) | **0.721**(0.050) |
| KIRP | **0.830**(0.067) | 0.761(0.090) | 0.817(0.090) |
| KICH | **0.780**(0.173) | 0.656(0.156) | 0.663(0.241) |
| LGG | **0.838**(0.033) | 0.786(0.039) | 0.817(0.046) |
| LIHC | 0.618(0.084) | 0.541(0.068) | **0.639**(0.044) |
| LUAD | 0.609(0.039) | 0.594(0.052) | **0.636**(0.043) |
| LUSC | 0.568(0.031) | 0.541(0.055) | **0.598**(0.045) |
| OV | 0.583(0.050) | 0.533(0.061) | **0.591**(0.040) |
| PAAD | 0.567(0.053) | 0.602(0.083) | **0.637**(0.021) |
| PRAD | 0.517(0.213) | 0.444(0.169) | **0.626**(0.218) |
| SKCM | 0.641(0.045) | 0.605(0.048) | **0.644**(0.043) |
| STAD | **0.588**(0.055) | 0.482(0.038) | 0.568(0.035) |
| THCA | 0.879(0.088) | 0.859(0.093) | **0.943**(0.021) |
| UCEC | 0.634(0.076) | 0.679(0.094) | **0.704**(0.059) |
| Average C-index | *0.658* | *0.618* | *0.678* |

**Section S4. Comparison with Cheerla and Gevaert (2019) using the modality combination of clinical and mRNA**

For a more comprehensive comparison with Cheerla and Gevaert (2019), we conducted another set of experiments using clinical, miRNA, and CNV modalities. The results are shown in **Figure S1** For all cancer types in the result, but OV and PAAD, our proposed model outperforms Cheerla and Gevaert (2019). This result further shows that the proposed model has a great improvement in prediction accuracy and stability compared with the previous work.

| 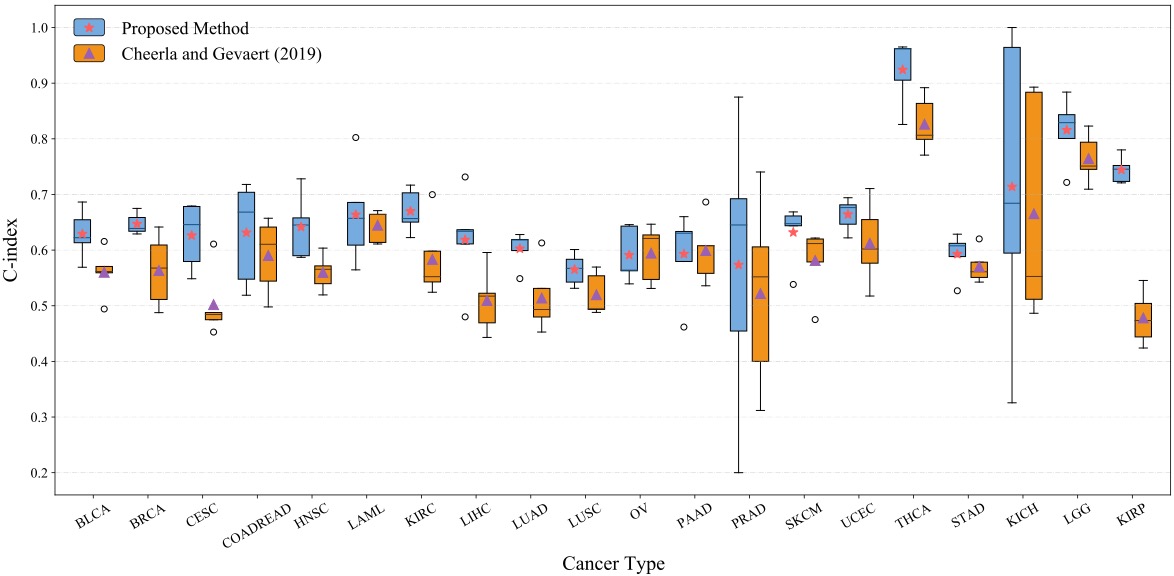 |
| --- |
| Fig. S1. C-index of the proposed model and the previous work (Cheerla and Gevaert, 2019) on the twenty cancer types using the modality combination of clinical, miRNA, and CNV. The proposed model outperforms the previous work on eighteen cancer types. |

**Reference**

1. A. Cheerla and O. Gevaert. Deep learning with multimodal representation for pancancer prognosis prediction. *Bioinformatics*, 35(14):446–454, 2019.
